# Managing supine hypertension in a patient with non-diabetic autonomic neuropathy receiving droxidopa for neurogenic orthostatic hypotension

**DOI:** 10.1007/s10286-017-0436-4

**Published:** 2017-06-16

**Authors:** Steven Vernino, Mark Lew, Daniel Kremens

**Affiliations:** 1 Department of Neurology and Neurotherapeutics, UT Southwestern, Dallas, TX USA; 2 Department of Neurology, University of Southern California, Los Angeles, CA USA; 3 Department of Neurology, Thomas Jefferson University, Philadelphia, PA USA

**Keywords:** Supine hypertension, Neurogenic orthostatic hypotension, Non-diabetic autonomic neuropathy, Droxidopa

## Challenge question

How does supine hypertension influence the management of orthostatic hypotension?

## Case presentation

Mr. L is a 65-year-old man who developed constipation, prickling feelings in his feet, and heat intolerance over the past several years, but is otherwise healthy. More recently, he has reported feeling dizzy and lightheaded upon standing, especially when first arising from bed in the morning. Orthostatic blood pressure (BP) measurements showed that he had marked orthostatic hypotension (OH) upon standing. His thorough evaluations also included autonomic testing and an endocrine work-up, which ruled out diabetes mellitus (which is a frequent cause of OH [1]). He was eventually diagnosed with non-diabetic autonomic neuropathy with neurogenic OH (nOH).

## Expert commentary (Dr. Vernino)

Damage to autonomic nerve fibers (autonomic neuropathy) can lead to symptoms of autonomic dysfunction including nOH. In most peripheral autonomic neuropathies, there are also symptoms of damage to peripheral “small fiber” sensory nerves that carry pain and temperature sensation (e.g., burning, prickling, or numbness in the feet). Other than diabetes, autonomic neuropathy can occur with amyloidosis, HIV infection, chronic alcohol exposure, certain toxins, and autoimmune disorders. Other autonomic neuropathies are idiopathic or inherited. Some of these disorders, notably amyloidosis, can also affect cardiac function, which complicates BP management.

## Case continuation

The patient was initially provided education about nOH and advised to use non-pharmacologic measures to control nOH. These included increasing fluid intake, liberalizing salt intake, elevating the head of the bed by at least 6–9 inches, and wearing compression garments including compression stockings and an abdominal binder. Despite these conservative measures, Mr. L remained symptomatic.

He was then prescribed droxidopa starting at 100 mg on a modified three times daily (TID) schedule (taken on awakening, at midday, and at least 3–4 h before bedtime) and up-titrated by an additional 100 mg TID every 24–48 h until symptomatic relief or until the maximum dosage of 600 mg TID is achieved. After reaching a steady droxidopa dosage of 200 mg TID, the patient noted resolution of orthostatic lightheadedness. However, he reported to his physician that his BP was very high in the evening when he lies down in bed. Supine BP at that time was 191/119 mmHg, and his seated BP was normal. This situation was indicative of supine hypertension, which is a frequent complication in patients with nOH [3].

## Expert commentary (Dr. Vernino)

Patients with autonomic failure need to understand that the loss of normal baroreflex function leads to nOH as well as supine hypertension, as the mechanisms that buffer the supine BP rise are impaired. Supine hypertension can generally be controlled by avoiding a supine posture. It is also important to take a sleep history from the patient to determine when they lie down in the evening and to ensure that they are not lying down for naps during the day. Pressor medications (such as droxidopa or midodrine) should not be taken close (within 4 h) to bedtime. If the patient does not have problems with nOH symptoms in the evening (nOH symptoms are usually worse in the morning), the last (afternoon/evening) dose of droxidopa can be reduced or eliminated.

## Expert commentary (Dr. Kremens)

Supine hypertension can be managed during the day by avoiding a supine posture at all. This is especially important in patients who are wearing compression garments or taking pressor medications (such as midodrine or droxidopa). Supine hypertension is very common in patients with nOH and can be a challenge in the management thereof. One strategy in patients taking droxidopa is first to make sure they are taking their last dose of droxidopa at least 4 h before sleep. If they are, it would be reasonable to consider decreasing the evening dose of droxidopa until supine hypertension resolves or, if necessary, withholding the final dose of the day. Sleeping with the head of the bed elevated at least 30°–45° will also reduce the risk of supine hypertension.

## Case continuation

As the patient reported having supine hypertension on his current regimen with droxidopa, he was instructed to reduce his final (evening) droxidopa dose to half. After making this change, the patient was instructed to measure his BP at home at specific times of the day. Measurements of BP in the sleeping position upon awakening (before morning medications) and in the evening (after lying in bed for at least 15 min) can be informative and reveal if supine hypertension remains significant. In addition, the patient was instructed to avoid lying flat while napping or at bedtime. Mr. L was also instructed to raise the head of the bed by 9 inches (c. 23 cm) by putting blocks or risers under the legs of the headboard. It was explained that using pillows to elevate the head is not sufficient or effective. In addition, the patient was instructed that he could have a glass of wine or eat a snack before bedtime with the explanation that these lifestyle measures can be useful to help reduce supine hypertension.

After 2 weeks of reducing his final droxidopa dose of the day by half and implementing the conservative measures described above, the patient reported that he continued to experience episodes of supine hypertension at bedtime. Supine BP at that time was 181/110 mmHg. To address his continued supine hypertension, the afternoon dose of droxidopa was eliminated, and dosing was adjusted to 200 mg twice daily, one dose at first waking and the second dose at noon. In addition, a small dose of short-acting antihypertensive medication, such as nifedipine, losartan, captopril, or transdermal nitroglycerin, at bedtime is sometimes necessary to control nighttime supine hypertension [2]. This patient was prescribed a transdermal nitroglycerin patch (0.1 mg/h) at bedtime to be removed upon waking in the morning.

## Case conclusion

Now, the patient reports that his symptoms of nOH remain resolved and he is no longer experiencing episodes of supine hypertension at bedtime.

## Expert commentary (Dr. Lew)

It is important to understand that patients with nOH can suffer not only from a significant drop in BP upon sitting or standing from a lying position but can also intermittently experience episodes of hypertension. This can make management of nOH with pharmacologic therapy even more complicated. A major potential adverse event of all medications used to treat nOH is supine hypertension and this needs to be aggressively managed. As in this case, decreasing the dose or discontinuing dose(s) of medications used to increase BP may be necessary. The use of short-acting antihypertensives may also be necessary in some patients if supine hypertension persists, despite discontinuing the presumed offending agents.
